# Prediction of Protein-Protein Interactions with Local Weight-Sharing Mechanism in Deep Learning

**DOI:** 10.1155/2020/5072520

**Published:** 2020-06-13

**Authors:** Lei Yang, Yukun Han, Huixue Zhang, Wenlong Li, Yu Dai

**Affiliations:** ^1^College of Computer Science and Engineering, Northeastern University, Shenyang, China; ^2^Key Laboratory of Intelligent Computing in Medical Image, Ministry of Education, Northeastern University, China; ^3^College of Software, Northeastern University, Shenyang, China

## Abstract

Protein-protein interactions (PPIs) are important for almost all cellular processes, including metabolic cycles, DNA transcription and replication, and signaling cascades. The experimental methods for identifying PPIs are always time-consuming and expensive. Therefore, it is important to develop computational approaches for predicting PPIs. In this paper, an improved model is proposed to use a machine learning method in the study of protein-protein interactions. With the consideration of the factors affecting the prediction of the PPIs, a method of feature extraction and fusion is proposed to improve the variety of the features to be considered in the prediction. Besides, with the consideration of the effect affected by the different input order of the two proteins, we propose a “Y-type” Bi-RNN model and train the network by using a method which both needs backward and forward training. In order to insure the training time caused on the extra training either a backward one or a forward one, this paper proposes a weight-sharing policy to minimize the parameters in the training. The experimental results show that the proposed method can achieve an accuracy of 99.57%, recall of 99.36%, sensitivity of 99.76%, precision of 99.74%, MCC of 99.14%, and AUC of 99.56% under the benchmark dataset.

## 1. Introduction

Protein plays an important role in the regulation of cell life activities, such as transcriptional regulation and signaling. At present, proteomics research with protein-protein interactions (PPIs) as the main research content is widely used in the field of medical drug target discovery. It is of great significance to promote the development of the biomedical industry.

Currently, PPI prediction research mainly uses the following two techniques: (1) experimental methods, including yeast two-hybrid [1, 2], protein chips [3, 4], coimmunoprecipitation [5], and Surface Plasmon Resonance (SPR) [6, 7]; (2) protein information-based computational methods, including protein primary sequence information [8] and spatial structure information of protein secondary structure [9–11]. Compared with the experimental method, the computational method has the advantages of fast verification speed and strong repeatability. Currently, with the development of the machine learning, some methods based on machine learning have been proposed. Currently, when using the machine learning to predict the PPIs, the methods always combine the two sequences of the protein together as the input [12, 13]. However, this may double the length of the feature vector and is prone to an overfitting problem. Besides, either a forward or a backward training method may sometimes overlook the long- and short-term effect which may result in the low prediction accuracy. In addition, the current methods always just consider 7 amino acids which may result in the low diversity of the features and the low accurate prediction performance.

In order to improve the prediction accuracy, the protein feature extraction and fusion method based on the combination of discrete wavelet transform and continuous wavelet transform is used to describe the protein amino acid sequence according to eight physicochemical properties of amino acid to improve the variety of the features to be considered in the prediction. Besides, with the consideration of the effect affected by the different input order of the two proteins, we propose a “Y-type” Bi-RNN model and use both the backward and forward training methods to train the model. In addition, in order to insure the training time caused on the extra training either a backward one or a forward one, this paper proposes a weight-sharing policy to minimize the parameters in the training. The experimental results verify that the method can effectively improve the accuracy and precision of the prediction results.

The following of the paper is organized as the follows: Section 2 introduces the related works of PPIs, Section 3 introduces the data and methods used in experiments, Section 4 introduces the experimental results and comparison with other methods in performance, and Section 5 introduces the conclusion of this paper and future work.

## 2. Related Work

Predicting PPIs using traditional experiments is often expensive and time-consuming, so many computational methods are used to infer PPIs from different sources of information, including phylogenetic profiles, tertiary structures, protein domains, and secondary structures [14, 15]. However, these approaches cannot be employed when prior knowledge about a protein of interest is not available.

With the rapid growth of protein sequence data, protein sequence-based prediction methods are becoming the most widely used PPI prediction tool. Therefore, many protein sequence-based methods have been developed to predict PPIs. Bock and Gough [16] used support vector machines (SVM) in conjunction with physicochemical descriptors to predict PPIs, proving that only protein sequence information is valid for predicting PPIs. Shen et al. [17] proposed a method for PPI prediction using only the amino acid sequence information of proteins. The method combines the kernel function and the combined ternary feature to describe the amino acid and obtains an accuracy of 83.90%. In 2010, Yu et al. [18] also used the primary structure of protein alone to deal with protein-protein interactions in unbalanced data. In 2013, You et al. [14] used amino acid sequence information and proposed ensemble extreme learning machines and principal component analysis methods to predict PPIs.

However, there are widespread problems of false-positive rate and false-negative rate in the above various experimental methods. In order to solve this problem, the researchers are more committed to the following two aspects to improve the accuracy of the prediction results. On the one hand, the robustness of feature extraction is improved to make the classification model effectively distinguish between positive and negative samples, thereby reducing the false-positive rate and false-negative rate. Thus, the dipeptide composition method [19, 20], the composition transformation distribution description method [21], the quasi-sequence-order descriptor (QSOD) [22], the wavelet transform [23], and other protein feature extraction methods are used in combination, such as the methods proposed by Du et al. [12], An et al. [24], and Huang et al. [25]. On the other hand, researchers hope to increase the complexity of the training model to increase the prediction results. For example, You et al. [26] use a random forest method. Xu et al. [27] used ensemble learning methods to predict PPIs. Li et al. [28] proposed a deep learning method DNN-PPI for CNN and LSTM. Although the above method has achieved certain effects, the training model takes too long, the model is too complicated, and the problem of overfitting the training model is also generated. Ref. [29] is to use the feature extraction and coding. The main work of us is to solve the problem of feature extraction and ensemble to get more appropriate features in order to ensure the accuracy. Besides, our work is to solve the problem of the effect caused by different input orders of the proteins. The extraction and coding method mentioned in the first reference can be used as our future work to improve the efficiency. The work of Ref. [30] takes the sequence of the two proteins as the input of the neural networks and uses a sLSTM to train the model. The work is similar to Li's work which is also referred in our manuscript. Such works' problem is that they do not consider the problem caused by the order of the two protein sequences. Ref. [31] adopts a Res2Vec to express the biological sequence and uses DNN to train the model, which also does not consider the relationship between the effect of the prediction and the order of the two protein sequences. But in the future work, we can use the proposed Res2Vec method to express the sequence also in order to avoid the overfitting problem. Ref. [32] is to use the RCNN to automatically select the features while the method does not consider the features in our work. Among the existing models, Wang et al. [13] proposed a method with a better comprehensive performance. They used discrete wavelet transform and continuous wavelet combination to extract protein features and obtained the accuracy of 97.38% under the yeast dataset. However, there are two shortcomings in this method. On the one hand, the method only considers the hydrophobicity of amino acids and regards hydrophobicity as the only property affecting protein interaction, ignoring other properties of amino acids. On the other hand, when using continuous wavelet transform to extract protein features, the time-frequency matrix is treated as an image, and the image is forcibly compressed to 60 × 60 pixels and then subjected to singular value extraction, thus forcing the compression of the image, which loses some of the information of the time-frequency matrix.

In order to improve the prediction accuracy, this paper proposes a feature extraction and fusion method. Besides, with the consideration of the effect affected by the different input order of the two proteins, a “Y-type” Bi-RNN model is proposed which uses both the backward and forward training methods to train the model. In addition, in order to insure the training time, this paper uses a weight-sharing policy to minimize the parameters in the training.

## 3. Materials and Methods

### 3.1. Datasets

#### 3.1.1. Benchmark Dataset

We got the original baseline data from the PPI dataset of PAN: http://http://www.csbio.sjtu.edu.cn/bioinf/LR_PPI/Data.htm[48]. The dataset contains 36,630 pairs of positive samples and 36,480 pairs of negative samples. Positive samples were obtained from the Human Protein Reference Database (HPRD) (2007 version). Negative samples (noninteraction pairs) are produced by paired proteins found at different subcellular locations. After eliminating the protein pairs of more than 1200 residue sequences, the benchmark dataset contains 29071 positive samples and 31496 negative samples. We randomly selected 6000 samples as the retention test set for model validation and the remaining samples as the training set. See Table 1 for details.

#### 3.1.2. Species Dataset

This paper selects five PPI datasets in the open source DIP database, the Human dataset, the *Saccharomyces cerevisiae* (*S. Cerevisiae*) dataset, the *Escherichia coli* (*E. coli*) dataset, the *Helicobacter pylori* (*H. pylori*) dataset, the *Caenorhabditis elegans* (*C. elegans*) dataset, and the *Mus musculus* (*M. musculus*) dataset.

*Human* dataset: in this work, the proposed method was verified with a high confidence PPI dataset. We collected this dataset from publicly available database of interacting proteins (DIP https://dip.doe-mbi.ucla.edu/), version 20170205. There are 37027 interactive pairs (positive sets) and 37027 noninteractive pairs (negative sets). The interacting pairs in this dataset were measured by the yeast two-hybrid assay in the DIP database. For the negative dataset, we followed the previous work [13, 27] and assumed that the proteins in different subcellular compartments do not interact with each other [33]. Specifically, the negative dataset was randomly generated from the Swiss-Prot database by excluding protein sequences which met the following conditions: (i) protein sequences without a certain subcellular location, (ii) protein sequences annotated with more than one subcellular location or “fragment” term, and (iii) protein sequences of less than 64 amino acids.

The interacting proteins of *S. cerevisiae*, *E. coli*, *H. pylori*, *C. elegans*, and *M. musculus* interacting proteins are derived from the DIP database. Among them, except for the dataset of *M. musculus*, the negative set generation method is the same as the *Human* dataset. The *M. musculus* dataset contains only positive samples for testing. The specific numbers are shown in Table 2.

### 3.2. Feature Extraction

Traditional feature extraction methods include dipeptide composition methods, composition conversion distribution description methods, and quasi-sequence-order descriptors (QSOD), among which the most commonly used amino acid physicochemical. The properties are hydrophobic *H*_1_, hydrophilic *H*_2_, side chain residue size *V*, polarity *P*_1_, polarizability *P*_2_, solvent accessible surface area SASA, and amino acid side chain net charge number NCI. Many previous studies have proved that it can effectively express protein characteristics. Xu et al. [27] selected the isoelectric point (PI) properties of amino acids for analysis, and the experimental results show that this physicochemical property has an important influence on the polypeptide chain. At the same time, Yu et al. [34] found that the amino acid's isoelectric point (PI) and ionization equilibrium constant (pKa) properties of the two amino acids can effectively express protein characteristics. Therefore, based on the physicochemical properties of the seven amino acids used in the conventional method, the isoelectric point (PI) and ionization equilibrium constant (pKa) of the amino acid were introduced for characterization. Considering the correlation between PI value and pKa value, this paper combines PI value and pKa value to obtain *P* value that can comprehensively measure amino acid and further extract protein sequence information according to *P* value. The specific calculation formula is as formula (1) and (2):

(1) $$\begin{array}{c} P=\text{PI}+u∙\text{pKa}, \end{array}$$

(2) $$\begin{array}{c} u=\frac{\sum_{n-1}^{20}\text{P}{\text{I}}_{n}}{\sum_{n-1}^{20}\text{p}{\text{Ka}}_{n}}. \end{array}$$

In summary, the physical and chemical properties of the 20 amino acids are shown in Table 3.

Next, standardize the above 8 physical and chemical properties according to formula (3):

(3) $$\begin{array}{c} P_{i,j}=\frac{P_{i,j}-P_{j}}{S_{j}}, \end{array}$$

wherein *P*_*i*,*j*_ represents the *j*^th^ physicochemical property of the *i*^th^ amino acid, *P*_*j*_ represents the mean of the *j*^th^ physicochemical property of 20 amino acids, and *S*_*j*_ represents the standard deviation of the *j*^th^ physicochemical property of 20 amino acids. Each protein amino acid sequence passes with hydrophobic *H*_1_, hydrophilic *H*_2_, side chain residue size *V*, polarity *P*_1_, polarizability *P*_2_, solvent accessible surface area SASA, amino acid side chain net charge number NCI, and *P*. The values of these eight attributes are converted into a sequence of numbers.

Wang et al. [15] proposed using wavelet to describe protein features and experimentally proved the feasibility of using wavelet transform for protein feature extraction. Discrete wavelet transform (DWT) is an implementation of the wavelet transform that uses discrete wavelet scale sets and translations and decomposes the input signal into mutually orthogonal wavelet sets. In this paper, the Dmeyer wavelet function is used in the discrete wavelet transform part, and Dmeyer is the discrete Meyer wavelet, which is used for the calculation of fast discrete wavelet transform.

This paper converts each amino acid sequence into a digital sequence by eight amino acid properties. By applying the DWT on any of these 8 digital sequences of a protein, each sequence-order vector is considered as a discrete time series and will put into one half-band high-pass filter and one half-band low-pass filter; then, the output sequence of the low-pass filter is then iterated four times to finally obtain 5 subsequences. In each subsequence, three kinds of data are extracted to reflect the internal information of the subsequence, which are (1) the average of the wavelet coefficients in each subsequence, (2) the standard deviation of the wavelet coefficients in each subsequence, and (3) the first four values with the largest absolute value in each subsequence and their relative positions; the relative position is calculated as

(4) $$\begin{array}{c} \text{location}=\frac{m}{n}, \end{array}$$

where *n* is the length of the current subsequence and *m* is the position in the sequence in which the current value is located.

In addition, this paper uses a 25-scale mexh continuous wavelet transform to transform each amino acid sequence. The Mexican hat “mexh” wavelet function is the second derivative of the Gauss function, with good regularity, large vanishing moment, and decomposition signal energy concentration. It has localized properties in the time domain and frequency domain. A matrix of *L* × 25 (*L* represents the length of the amino acid sequence) can be extracted by the mexh continuous wavelet transform, and the vector features of the 25 dimensions are extracted according to the decomposition of the singular value matrix. Eventually, each protein sequence is converted into a feature vector of 600 dimensions.

### 3.3. Deep Neural Network with Local Weight Sharing

Currently, some works have been proposed to predicting PPIs based on neural networks, such as Du et al. [12] and Li et al. [28]. When using a neural network model to predict protein interactions, you can reduce the impact of protein input order on the prediction result by entering two protein features and training separately. This method of inputting proteins can coordinate protein features and protein interactions. The relationship between the feature characteristics improves the overall prediction result. However, neural network models often require too many training parameters, resulting in redundant training time. In this paper, considering the influence of protein input order on prediction results, a deep neural network with local weight sharing is proposed. The network model adopts a “Y-type” neural network model, including a weight-sharing Bi-RNN layer, a buffer layer, and a dense layer, wherein the weight-sharing Bi-RNN layer can reduce the influence of the protein on the predicted input order and accelerate the training. The weight-sharing Bi-RNN to the same parameter that needs to be learned on the Bi-RNN layer on both sides, that is, the parameter values that need to be learned in the corresponding positions on both sides of the layer, are the same, thereby improving the accuracy of the model prediction result and the model training speed.

**Figure 1:**
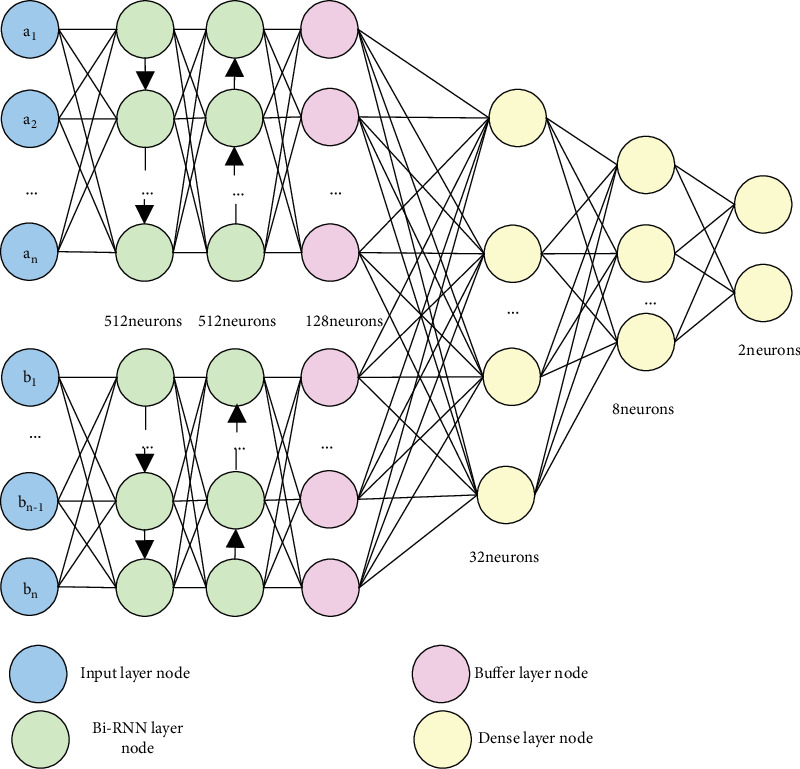
“Y-type” Bi-RNN model diagram of local weight sharing.

The neural network model of local weight sharing is shown as Figure 1. The input layer is divided into two parts containing 1200 neurons, half of which is for the 600 features of protein A and the other half of which is for the 600 features of protein B. The layer of BI-RNN is also divided into 2 parts, respectively, for proteins a and b. Each part has 2 layers. Each of the layer contains 512 neurons. Then, the total number of neurons in the BI-RNN layer is 512∗2∗2 = 2048. The next layer is buffer layer whose function is to connect the parts of a and b into the dense layer for training. In the buffer layer, there will be totally 256 neurons for the proteins a and b which means there will be 256 features to be used. The last layer is the dense layer which contains 3 layers with 32, 8, and 2 neurons in each layer, respectively. The layer with 2 neurons is the last layer in the dense layer which is used to output the classification result. Here, the output (1,0) means there exists interaction, and (0,1) otherwise.

This paper uses the ReLU activation function. Since the ReLU activation function will make the output of a part of neurons zero, the network has a certain sparsity, which reduces the interdependence of parameters to a certain extent, and thus effectively avoids overfitting. The ReLU activation function is used in the model, and the ReLU activation function expression is as

(5) $$\begin{array}{c} \text{ReLU}\left(x\right)=\left\{\begin{array}{c} 0,x\leq 0,\\ x,x>0. \end{array}\right. \end{array}$$

**Figure 2:**
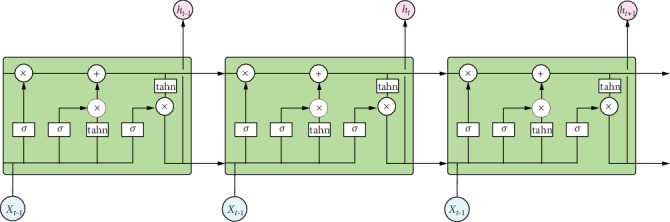
Schematic diagram of LSTM neurons.

RNN can be regarded as a neural network for information transmission in time series. The depth of the model corresponds to the length of the sequence. The gradient disappearance problem that often occurs in the neural network appears correspondingly in the time dimension of the RNN. In order to solve the gradient dispersion problem of RNN in the time dimension, the researchers proposed the long and short memory unit LSTM, which proved that LSTM is very effective in solving long sequence dependence problems. However, since the use of RNN will cause the gradient to disappear, in order to solve this problem, this paper uses Long Short-Term Memory (LSTM) [35, 36]; the neurons are as Figure 2.

LSTM is a variant model of RNN. The parameters of an LSTM neuron include input gate, forgetting gate, output gate, and unit and unit input activation vectors, which are represented by *i*_*t*_, *f*_*t*_, *o*_*t*_, and *C*_*t*_, respectively. The specific expression is as shown in formula (6)–(11), where *h*_*t*−1_ is the output of the previous cell and *x*_*t*_ is the input of the current cell. *σ* represents the sigmoid function.

(6) $$\begin{array}{c} f_{t}=\sigma \left(W_{f}∙\left[h_{t-1},x_{t}\right]+b_{f}\right), \end{array}$$

(7) $$\begin{array}{c} i_{t}=\sigma \left(W_{i}∙\left[h_{t-1},x_{t}\right]+b_{i}\right), \end{array}$$

(8) $$\begin{array}{c} \tilde{C}=\tanh\left(W_{C}∙\left[h_{t-1},x_{t}\right]+b_{C}\right), \end{array}$$

(9) $$\begin{array}{c} C_{t}=f_{t}\times C_{t-1}+i_{t}\times \tilde{C_{t}}, \end{array}$$

(10) $$\begin{array}{c} o_{t}=\sigma \left(W_{o}\left[h_{t-1},x_{t}\right]+b_{o}\right), \end{array}$$

(11) $$\begin{array}{c} h_{t}=o_{t}\times \tanh\left(C_{t}\right). \end{array}$$

### 3.4. Forward and Backward Model Training

In the construction of protein sequences, the traditional method uses the way of binding *P*_*A*_ = {*a*_1_, *a*_2_, ⋯*a*_*n*_} and *P*_*B*_ = {*b*_1_, *b*_2_, ⋯*b*_*n*_} sequentially to construct PPI characteristics (*n* represents the number of features described by the protein), as

(12) $$\begin{array}{c} P_{\text{PPI}}=P_{A}⊕P_{B}=a_{1},a_{2},\cdots a_{n},b_{1},b_{2},\cdots b_{n}. \end{array}$$

**Figure 3:**
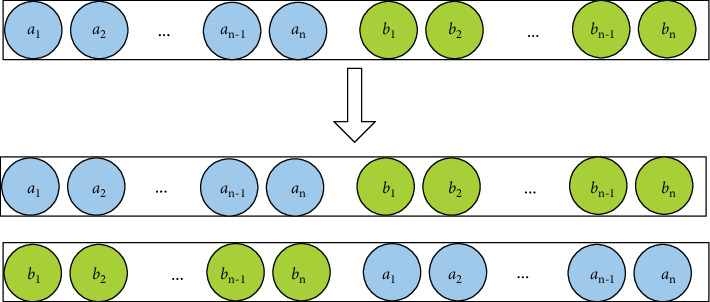
Schematic diagram of forward and backward model training process.

In view of the problem that the classification model is easy to overfitting and the order of protein feature binding affects the results, this paper proposes a combination of forward and backward protein feature sequences. The improvement based on the training method is based on the use of the above formula to construct the PPI characteristics. On the basis of the training model in the sequential combination, the PPI feature A in the training set is reinversely combined into B and added to the training set, so that the number of training sets of the training sample reconstruction is doubled and the number of test sets remains the same, as shown in Figure 3.

Every protein interaction data record can be expressed as (*a*_1_, *a*_2_, ⋯*a*_600_, *b*_1_, *b*_2_, ⋯*b*_600_(*l*_1_, *l*_2_)), where *a* represents the features of protein A, *b* represents the features of protein B, and (*l*_1_*,l*_2_) represents the label. The data of the labels are (1,0) and (0,1). (1,0) indicates that there exists an interaction relationship, and (0,1) means not. In the environment of python3.5, TensorFlow1.12.0 can be used to build a neural network according to the method in the paper. The batch_size is set to 128, learning_rate is set to 0.05, and trains are set for 200 rounds.

## 4. Experimental Results and Comparison

### 4.1. Evaluation Measures

In this experiment, we used a fivefold cross-validation process to prevent the calculation method from overfitting on the dataset or misjudging the result due to unbalanced data extraction. The evaluation criteria for the algorithm use several widely used parameters: accuracy, recall, specificity, precision, and MCC (Mathew's correlation coefficient). Some of the above parameters are defined as formula (13)–(17):

(13) $$\begin{array}{c} \text{Accuracy}=\frac{\text{TP}+\text{TN}}{\text{TP}+\text{TN}+\text{FP}+\text{FN}}, \end{array}$$

(14) $$\begin{array}{c} \text{Recall}=\frac{\text{TP}}{\text{TP}+\text{FN}}, \end{array}$$

(15) $$\begin{array}{c} \text{Specificity}=\frac{\text{TN}}{\text{TN}+\text{FP}}, \end{array}$$

(16) $$\begin{array}{c} \text{Precision}=\frac{\text{TP}}{\text{TP}+\text{FP}}, \end{array}$$

(17) $$\begin{array}{c} \text{MCC}=\frac{\text{TP}\times \text{TN}-\text{FP}\times \text{FN}}{\sqrt{\left(\text{TP}+\text{FP}\right)\left(\text{TP}+\text{FN}\right)\left(\text{TN}+\text{FP}\right)\left(\text{TN}+\text{FN}\right)}}, \end{array}$$

where the true-positive (TP) value denotes the number of true samples which are predicted correctly; the false-negative (FN) value is the number of true samples predicted to be noninteracting pairs incorrectly; the false-positive (FP) value is the number of true noninteracting pairs predicted to be PPIs falsely; and the true-negative (TN) value is the number of true noninteracting pairs predicted correctly.

### 4.2. Training and Validation on the Benchmark Dataset

We randomly divide the training set into five sets by fivefold cross-validation, and cross-train. Finally, the best performance model is tested under the hold-out test set. The specific data are shown in Table 4. We can see that the accuracy of the fivefold cross-validation is almost the same. For testing the hold-out set, the model for test set 2 is used which achieved an accuracy of 99.57%, recall of 99.36%, sensitivity of 99.76%, precision of 99.74%, MCC of 99.14%, and AUC of 99.56%.

To further confirm the effectiveness of the model, we compare the experimental results by constantly switching the ratio of training set to test set, as shown in Table 5. We can see that the model can maintain a robust effect in different proportions.

### 4.3. Multispecies Training Evaluation

To further explore the validity of the model, we used the same method to conduct experiments on different species datasets. Similarly, the training sets of each species were randomly divided into five sets for cross-training, and the best model was selected for testing.  Table 6 shows the results. In each training set, the performance remains robustness, and the data fluctuation does not exceed 1%. Finally, we can see that the accuracy in E. coli, C. elegans, S. cerevisiae, and human is 95.04%, 98.14%, 99.96%, and 99.94%, respectively.

### 4.4. Compare the Performance of Feature Extraction and Fusion

**Figure 4:**
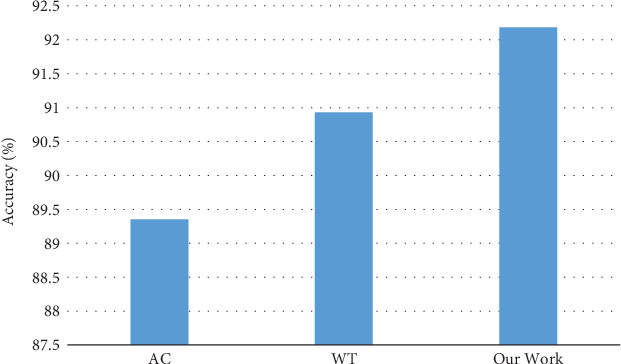
Experimental results of different feature extraction and fusion methods in a support vector machine.

In order to verify the feature extraction and fusion, under the same dataset, compare the performance of the feature extraction and fusion in our work and the works (signified as AC) which uses the amino acid composition based on position information to extract the features, as well as the works (signified as WT) which uses the feature extraction method based on multiattribute wavelet transform. For just comparing the performance of the feature extraction and fusion, we use the SVM classifier to train the model. The result can be seen in Figure 4. From the result, we can see that our work has a higher accuracy than others.

### 4.5. Comparison with Other Methods

In this section, we compare the performance of the proposed method with other different methods, by using the S. cerevisiae dataset, as shown in Table 7. We can see that in the counterpart methods, the accuracy is between 83.35% and 98.78%, most of them have exceeded 90%, and the highest accuracy rate is 98.78%. Compared with our method, except that precision in Ref. [28], the other attributes are higher than other methods, the minimum difference of accuracy is 1.16%, the minimum difference of sensitivity is 1.72%, the minimum difference of precision is 0%, and the minimum difference of MCC is 1.29%.

### 4.6. Comparison Time Performance with Other Deep Learning Methods

Based on the same machine configuration, this paper also compares the training time required for different training models.  Table 8 lists the comparison of the training time required between different algorithms. It can be seen that the training time of the partial-input two-way cyclic neural network shared by local weights is almost the same as that of the five-layer fully connected neural network under the same-order neurons. At the same time, compared with the DeepPPI training model proposed by Du et al. [12], the training speed is increased by 70 seconds, which is 251 seconds higher than that of the deep neural network without local weight sharing.

### 4.7. Training Other Models by Using the Features of Forward and Backward Reconstruction

The experimental results are shown in Table 9. We can see that the training methods based on the characteristics of forward and backward training can improve the performance of Xu's method which can improve the accuracy by 1.26%, recall by 1.98%, sensitivity by 0.99%, precision by 2.13%, and MCC by 2.91%.

### 4.8. Cross-Species Cross-Validation Evaluation

According to Table 10, we can see that when the training set is human, the test results are better, most of which are more than 90 percent. However, other species can only play a certain role in the M. musculus dataset for testing. For data analysis, since human datasets are almost the sum of other kinds of datasets, robust models can be effectively trained. The best is 98.39%.

## 5. Conclusion and Future Work

The protein feature extraction and fusion method based on the combination of discrete wavelet transform and continuous wavelet transform is used to describe the protein amino acid sequence to improve the variety of the features to be considered in the prediction. When constructing protein interaction features, the common method is to directly combine the two protein features in sequence. However, this method of constructing interaction features doubles the length of the feature vector, which is likely to cause overfitting under limited protein interaction datasets. At the same time, because two interacting protein features are equally important, constructing mutual features of proteins is likely to result in different predictions due to different binding sequences. In this paper, based on the use of sequential binding protein features, the forward and backward model training methods are proposed to address this issue. Besides, a weight-sharing policy is proposed to minimize the parameters in the training in order to insure the training time. This paper defines protein interactions as a binary classification problem. In the future, we can consider whether the problem can be mapped to a multiclassification problem where the prediction result can be a certain type of interaction. Besides, currently, expressing the sequence by using an appropriate way can be used to solve the overfitting problem. Then, in the future, the coding method for expressing the features will be studied.

## Figures and Tables

**Table 1:** Benchmark dataset.

Dataset	Positive samples	Negative samples	Total
Benchmark set	29071	31496	60567
Training set	26128	28439	54567
Hold-out test set	2943	3057	6000

**Table 2:** Species dataset.

Species dataset	Positive samples	Negative samples	Total
*Human*			
Original set	37027	37027	74054
Training set	29622	29622	59244
Testing set	7405	7405	14810
*S. cerevisiae*			
Original set	5943	5943	11886
Training set	4754	4754	9508
Testing set	1189	1189	2378
*E. coli*			
Original set	6954	6954	13908
Training set	5023	5023	10046
Testing set	1931	1931	3862
*C. elegans*			
Original set	4030	4030	8060
Training set	3224	3224	6448
Testing set	806	806	1612
*H. pylori*			
Original set	1458	1458	2916
Training set	1116	1116	2332
Testing set	342	342	684
*M. musculus*			
Original set	22683	—	22683

**Table 3:** Physicochemical properties of 20 amino acids.

Code	*H*_1_	*H*_2_	*V*	*P*_1_	*P*_2_	SASA	NCI	*P*
A	0.62	-0.5	27.5	8.1	0.046	1.181	0.007187	12.772
C	0.29	-1	44.6	5.5	0.128	1.461	-0.03661	10.4312
D	-0.9	3	40	13	0.105	1.587	-0.02382	8.4134
E	-0.74	3	62	12.3	0.151	1.862	0.006802	9.1455
F	1.19	-2.5	115.5	5.2	0.29	2.228	0.037552	11.6877
G	0.48	0	0	9	0	0.881	0.179052	12.742
H	-0.4	-0.5	79	10.4	0.23	2.025	-0.01069	12.669
I	1.38	-1.8	93.5	5.2	0.186	1.81	0.021631	12.5099
K	-1.5	3	100	11.3	0.219	2.258	0.017708	15.9477
L	1.06	-1.8	93.5	4.9	0.186	1.931	0.051672	12.4699
M	0.64	-1.3	94.1	5.7	0.221	2.034	0.002683	11.6655
N	-0.78	2	58.7	11.6	0.134	1.655	0.005392	11.3355
P	0.12	0	41.9	8	0.131	1.468	0.239531	11.9434
Q	-0.85	0.2	80.7	10.5	0.18	1.932	0.049211	11.8677
R	-2.53	3	105	10.5	0.291	2.56	0.043587	15.839
S	-0.18	0.3	29.3	9.2	0.062	1.298	0.004627	11.8877
T	-0.05	-0.4	51.3	8.6	0.108	1.525	0.003352	12.0855
V	1.08	-1.5	71.5	5.9	0.14	1.645	0.057004	12.1677
W	0.81	-3.4	145.5	5.4	0.409	2.663	0.037977	12.662
Y	0.26	-2.3	117.3	6.2	0.298	2.368	0.023599	11.8677

**Table 4:** Performances of deep neural network with local weight sharing.

Test set	Accuracy (%)	Recall (%)	Sensitivity (%)	Precision (%)	MCC (%)	AUC (%)
1	99.88	99.87	99.88	99.87	99.75	99.88
2	99.88	99.75	100.00	100.00	99.75	99.87
3	99.57	99.21	99.88	99.87	99.14	99.55
4	99.88	99.13	99.88	99.87	99.02	99.50
5	99.82	99.62	100.00	100.00	99.63	99.81
Hold-out	99.57	99.36	99.76	99.74	99.14	99.56

**Table 5:** Performances with different proportions of training and testing sets.

Train/test	Accuracy (%)	Recall (%)	Sensitivity (%)	Precision (%)	MCC (%)	AUC (%)
0.3/0.7	99.71	99.07	99.84	99.83	98.94	99.46
0.25/0.75	99.95	100.00	99.90	99.90	99.90	99.95
0.2/0.8	99.88	99.87	99.88	99.87	99.75	99.88
0.1/0.9	99.75	99.49	100.00	100.00	99.51	99.74

**Table 6:** Performance comparisons on datasets for other species.

Species	Test set	Accuracy (%)	Recall (%)	Sensitivity (%)	Precision (%)	MCC (%)	AUC (%)
*E. coli*	1	95.29	91.22	99.35	99.29	90.88	95.29
	2	95.40	91.65	99.14	99.07	91.05	95.39
	3	95.40	91.94	98.85	98.76	91.01	95.39
	4	95.04	90.71	99.35	99.29	90.41	95.03
	5	95.22	91.07	99.35	99.29	90.75	95.21
	Hold-out	95.04	90.71	99.35	99.29	90.41	95.03

*C. elegans*	1	98.33	96.81	99.76	99.74	96.68	98.28
	2	98.20	96.56	99.76	99.74	96.44	98.16
	3	98.14	96.43	99.76	99.74	96.32	98.09
	4	98.33	97.19	99.40	99.35	96.67	98.30
	5	98.45	97.32	99.52	99.48	96.92	98.42
	Hold-out	98.14	96.81	99.40	99.35	96.30	98.10

*S. cerevisiae*	1	99.94	99.83	100.00	100.00	99.87	99.92
	2	99.99	99.97	100.00	100.00	99.98	99.99
	3	99.88	99.83	99.91	99.83	99.74	99.87
	4	99.99	99.97	100.00	100.00	99.98	99.99
	5	99.96	99.92	99.98	99.97	99.91	99.95
	Hold-out	99.86	99.61	100.00	100.00	99.70	99.80

*Human*	1	99.93	99.88	99.97	99.97	99.85	99.93
	2	99.92	99.86	99.97	99.97	99.84	99.92
	3	99.87	99.84	99.91	99.90	99.74	99.87
	4	99.89	99.84	99.93	99.93	99.77	99.88
	5	99.86	99.82	99.91	99.90	99.73	99.86
	Hold-out	99.94	99.88	100.00	100.00	99.88	99.94

**Table 7:** Comparison with other methods.

Method	Accuracy (%)	Sensitivity (%)	Precision (%)	MCC (%)
Our work	99.57	99.76	99.74	99.14
Work in Ref. [28]	98.78	98.23	98.61	97.57
Work in Ref. [13]	97.38	94.76	100.00	94.89
Work in Ref. [12]	94.43	96.65	94.38	88.97
Work in Ref. [26]	93.92	91.10	96.45	88.56
Work in Ref. [24]	92.65	92.63	92.67	86.40
Work in Ref. [25]	92.05	88.82	95.87	86.09
Work in Ref. [36]	89.33	88.87	89.93	N/A
Work in Ref. [27]	83.35	92.95	83.32	63.77

**Table 8:** Comparison time performance with other deep learning methods.

Method	Our work	5-layer fully connected neural network	DeepPPI [12]	Deep neural network without local weight sharing
Time (seconds)	312	309	382	563

**Table 9:** Training other models by using the features of forward and backward reconstruction.

Method	Test set	Accuracy (%)	Recall (%)	Sensitivity (%)	Precision (%)	MCC (%)
Xu et al.'s work [27]	1	84.77	67.50	94.06	85.95	65.78
	2	84.88	67.53	94.04	85.68	65.78
	3	84.88	67.68	94.53	85.33	65.47
	4	84.85	68.01	93.65	84.85	65.56
	5	84.57	66.41	94.07	85.42	64.93
	Average	84.79	67.43	94.07	85.45	65.50
	Average before improvement	83.35	65.45%	92.95	83.32	63.77

**Table 10:** Performances on the cross-species validations.

Training set	Test set	Accuracy (%)
*Human*	*M. musculus*	98.39
	*C. elegans*	95.75
	*S. cerevisiae*	91.11
	*H. pylori*	86.15
	*E. coli*	50.20

*C. elegans*	*M. musculus*	96.23
	*S. cerevisiae*	55.00
	*H. pylori*	52.97
	*E. coli*	50.19
	*Human*	49.72

*S. cerevisiae*	*M. musculus*	97.23
	*C. elegans*	55.32
	*E. coli*	51.19
	*Human*	49.72
	*H. pylori*	65.97

*E. coli*	*M. musculus*	95.23
	*C. elegans*	51.66
	*S. cerevisiae*	50.24
	*Human*	44.12
	*H. pylori*	43.81

## Data Availability

The data supporting the conclusions are presented in the main article. The code can be found in https://github.com/joddiedai/ppi.
